# A New Morbific Germ Discovered

**Published:** 1887-10

**Authors:** Frank S. Billings

**Affiliations:** Director; Patho-Biological Laboratory, State University, Lincoln, Neb.


A NEW MORBIFIC GERM DISCOVERED.–A scientific worker in Nebraska announces the discovery of the morbic germ of Texas fever. As to the validity of his claim we are unable to judge. He has been kind enough to forward us specimens of his germs, and which the microscope shows to correspond to his description. Experts in bacteriology will soon confirm or refute his claims. It would seem odd if the wilds of the West were able to make a real scientific discovery, after the uncomplimentary descriptions given of the western doctors by their brethren of the Atlantic cities.

But we give entire the writer's own description of his discovery:

THE GERM OF TEXAS FEVER.

*Editor of the Americzn Lancet:*

My Dear Sir.—I wish to announce to the medical profession and scientific laboratories, the first discovery of the germ of that devastating American cattle plague, the Texas, or Southern cattle fever. In order to be justified in making such an announcement, an investigator must fulfil the following conditions:

1. Find a given germ invariably in the organs of organisms having a specific disease.

2. Isolate and cultivate said organisms from these tissues.

3. Prove that said germ has pathogenetic action upon animals used for experimentation.

4. Produce the same disease in healthy animals of the same species in which the disease occurs under natural circumstances.

These conditions I have fulfilled in every particular.

First, by finding the germ in the blood, gall, urine, liver, spleen, and kidneys.

Second, in making pure cultures from the blood and liver.

Third, by killing ground squirrels with inoculations of pure cultures in about 48 hours.

Fourth, by inducing the same disease in cattle and proving the presence of the germ by microscopic examination and cultivation.

This germ belongs to that class of septic germs represented by our swine plague, the "wild seuche" and "schwino seuch" of Germany, and rabbit septicæmia. It is a bacterium. It colors at its poles and has a clear or non-coloring middle piece to its body. Like the H. C. germ, it has motility in hanging drop cultures and also in the blood serum from the original blood of a diseased animal. It cannot be distinguished from the H. C. germ under the microscope, or in its growth on agar-agar, but differentiates itself very sharply by its growth on potatoes, having a delicate straw color, while the H. C. germ has a muddy, coffee color—"boarding house coffee color," a friend suggests. In gelatin I have not yet tested it, on account of the obstacles of the prevailing temperature.

That it is the first discovery of this germ, may be seen by reference to the literature. In the Agricultural Reports of the Department at Washington is recorded all that has been done on this subject. In those of 1880–81 and 1884, Ditmers reports and illustrates a "bacillus," and a gigantic one at that. It is not this germ. In the Report of 1883, Salmon claims to have discovered a "diplococcus," which has "a figure 8 form," and is "without any power of movement." Neither give any experimental evidence of a positive character to support their conclusions. The same will, however, follow this announcement in course of time.

I remain, my dear sir, yours truly,

FRANK S. BILLINGS, Director.

PATHO-BIOLOGICAL LABORATORY, STATE UNIVERSITY,
LINCOLN, Neb., Sept. 16, 1887.

—*American Lancet.*

---

THF FIRST DISCOVERY, OR THE DISCOVERY, FIRST ?

Dr. F. S. Billings, (*American Lancet*) claims the "first discovery of the germ of the Texas, or Southern Cattle fever." We wonder who will make the second or next "discovery" ? We have always thought that a "discovery" was *essentially* "first"—the first sight of anything.

The *Lancet* says : "It would seem odd, if the wilds of the West were able to make a real scientific discovery, after the uncomplimentary descriptions given of the western doctors by their brethren of the Atlantic cities."

Whether or not Texas may be considered in the "wilds of the West." we beg to remind "our esteemed" that Dr. J. W. McLaughlin, of Austin, two years ago, discovered, isolated, cultivated and demonstrated the "germ" of *Dengue,*—and received credit at last, for a scientific discovery.

---

"THE TEMPTING FRUIT OF THE HONEY LOCUST [ !] The *Maryland Medical Jourual* speaks of "the tempting fruit" of the thorn locust, after it is "fully tinged by the autumn frosts," and "*still further shrivelled up*" [*!*] by the chilling blast," etc. Surely this is a "Goak" as Artemus Ward would say : the "niggers" use it to make "beer,"—the only persons who were ever known to "esteem" it; it is about as "luscious" as a tooth pick.

